# A Hundred Years of Bacteriophages: Can Phages Replace Antibiotics in Agriculture and Aquaculture?

**DOI:** 10.3390/antibiotics9080493

**Published:** 2020-08-07

**Authors:** Carmen Sieiro, Lara Areal-Hermida, Ángeles Pichardo-Gallardo, Raquel Almuiña-González, Trinidad de Miguel, Sandra Sánchez, Ángeles Sánchez-Pérez, Tomás G. Villa

**Affiliations:** 1 Department of Functional Biology and Health Sciences, Microbiology Area, University of Vigo, Lagoas-Marcosende, 36310 Vigo, Spain; Lara.areal.hermida@uvigo.es (L.A.-H.); maria.angeles.pichardo.gallardo@uvigo.es (Á.P.-G.); ralmuina@alumnos.uvigo.es (R.A.-G.); 2 Department of Microbiology and Parasitology, University of Santiago de Compostela, 5706 Santiago de Compostela, Spain; trinidad.demiguel@usc.es (T.d.M.); sandra.sanchez@usc.es (S.S.); 3 Sydney School of Veterinary Science, Faculty of Science, University of Sydney, Sydnay NSN 2006, Australia; angelines2085@icloud.com

**Keywords:** agriculture, aquaculture, bacterial diseases, phages, phage therapy, biocontrol, antibiotic-resistant bacteria

## Abstract

Agriculture, together with aquaculture, supplies most of the foodstuffs required by the world human population to survive. Hence, bacterial diseases affecting either agricultural crops, fish, or shellfish not only cause large economic losses to producers but can even create food shortages, resulting in malnutrition, or even famine, in vulnerable populations. Years of antibiotic use in the prevention and the treatment of these infections have greatly contributed to the emergence and the proliferation of multidrug-resistant bacteria. This review addresses the urgent need for alternative strategies for the use of antibiotics, focusing on the use of bacteriophages (phages) as biocontrol agents. Phages are viruses that specifically infect bacteria; they are highly host-specific and represent an environmentally-friendly alternative to antibiotics to control and kill pathogenic bacteria. The information evaluated here highlights the effectiveness of phages in the control of numerous major pathogens that affect both agriculture and aquaculture, with special emphasis on scientific and technological aspects still requiring further development to establish phagotherapy as a real universal alternative to antibiotic treatment.

## 1. What Are Bacteriophages?

**Figure 1:**
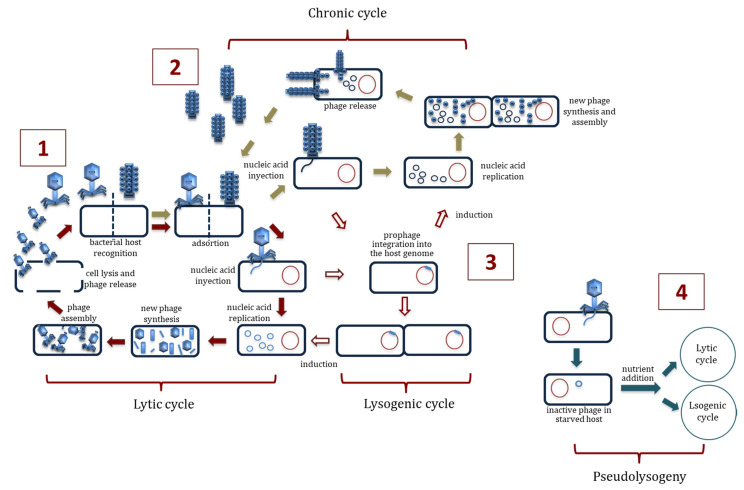
Phage–host interactions. (**1**): Lytic cycle. (**2**): Chronic cycle. (**3**): Lysogenic cycle. (**4**): Pseudolysogeny.

Bacteriophages, also known informally as phages (from the Greek word “phagein”, which means “to devour”), are viruses with the ability to infect and kill bacteria; hence, the term “bacteriophages” means “bacteria eaters”. Phages are ubiquitous; they are present in all terrestrial and aquatic habitats where their host bacteria live, controlling those bacterial populations. Bacteriophages are the most abundant biological forms in the biosphere, with an estimated number of 10^31^ [1]. Phage taxonomic classification is the responsibility of the Bacterial and Archaeal Viruses Subcommitee (BAVS) of the International Committee on the Taxonomy of Viruses (ICTV), which have extended the formal virus classification to 15 hierarchical ranks. The system has evolved from the one based on the morphology and the molecular composition of the virus genome (the main criteria for the classification at the family level) to the current system that also considers host range, pathogenity, and sequence similarity. ICTV currently defines 19 phage families, the most-well characterized being *Myoviridae*, *Siphoviridae*, *Podoviridae*, *Inoviridae*, *Microviridae* and the recently described ones *Ackermannviridae* and *Herelleviridae*, all of them within the *Caudovirales* order [2,3,4,5]. Phages, composed of a capsid that encloses the viral genome consisting of either single or double-stranded DNA or RNA [2], are classified as either virulent or temperate according to their life cycle. After infection, virulent phages take control of the metabolic machinery of the bacteria and use it to replicate themselves and synthesize new phage particles. The viral progeny is released from the host cell by lysis, resulting in the death of the host and allowing the new particles to start a new lytic cycle. Temperate phages, on the other hand, often initiate a lysogenic cycle; this involves the integration of the viral nucleic acid into the bacterial genome, remaining in the prokaryotic cell as prophages. These prophages are transmitted, together with the bacterial genome, to the descendants of the host, and this transmission continues until the lytic cycle is induced. A variant of the lysogenic cycle is the so-called carrier state or pseudolysogenic cycle, in which the nucleic acid of the phage does not replicate but instead remains inactive within the host. Probably, pseudolysogeny occurs when cells are undergoing starvation, and there is not enough available energy for viral gene expression. When nutrients are again provided, the pseudolysogenic state is resolved with either the initiation of the lytic cycle or the establishment of true lysogeny. Finally, another form of phage–host cell interaction is referred to as chronic infection. In this type of lifecycle, the phage replicates actively in the host originating the viral progeny that exit the bacteria by different mechanisms without bacterial lysis (Figure 1) [6,7]. The ability of bacteriophages to kill bacteria advocates a widespread role for phages as an alternative to antibiotics. The use of lytic phages or their products for the treatment of bacterial diseases is known as phage therapy [8], and this type of therapy presents major advantages over chemotherapy (Box 1).

Box 1 **Advantages of phage therapy.**

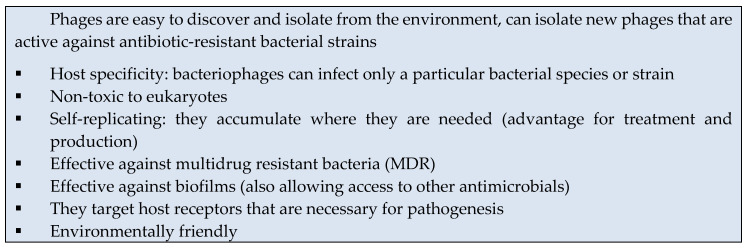

## 2. A Look at the Past

In 1896, Ernst Hankin demonstrated the presence of antimicrobial activity against *Vibrio cholera* in the waters of the Ganges river in India [9]. However, it was not until 1915–1917 that Twort and d’Herelle, independently, described the putative existence of filterable and transmissible agents with the ability to lyse bacteria [10,11,12]. According to Twort, a British pathologist, the lytic principle would be of enzymatic nature, while d’Herelle, a Canadian microbiologist, speculated that it represented a virus. However, it took an additional 30 years for the hypothesis formulated by d’Herelle to be confirmed; the potential of phages as antimicrobials was soon suspected and quickly corroborated. D’Herelle demonstrated in 1919 that phage preparations could be used to treat patients with dysentery, at the *Hospital des Enfants-Malades* in Paris [13]. Before treating patients with phage preparations, d’Herelle tested the safety of the treatment on himself. Subsequently, numerous studies demonstrated the effectiveness of phages treatment against a variety of diseases, including cholera, staphylococcal infections, typhoid fever, and *Shigella* and *Salmonella* colitis [14,15,16]. The treatment was so effective that it prompted many pharmaceutical companies to start marketing phage preparations to combat bacterial infections. Almost in parallel, researchers started evaluating the possibility of treating plant bacterial diseases with phages [17,18]. In 1924, Mallman and Hemstreet demonstrated that a filtrate, collected from decaying cabbages, inhibited in vitro the development of the bacterium *Xanthomonas campestris* pv. *campestris*, a microorganism that causes black rot in these crucifers [17]. Subsequently, a variety of phages effective against different phytopathogenic bacteria, including *Pectobacterium carotovorum* subsp. *carotovorum* [19], *Pectobacterium atrosepticum* (formerly *Erwinia carotovora* subsp. *atroseptica*) [20] or *X. malvacearum* [21], were isolated. Despite the early success of bacteriophages as antimicrobials, their use as a treatment, and even phage research, declined dramatically with the discovery of antibiotics, penicillin in particular. Only some Eastern European nations, such as Poland, Georgia, and Russia, continued the research in this field and the treatment of infectious diseases with phage therapy [14,22,23]. Unfortunately, despite the demonstrated potential for phage treatment of phytopathogenic bacteria, some researchers remained skeptical and questioned the efficacy of phage therapy in this field [24]. All of this, in combination with the perception, particularly among clinicians, that antibiotic treatment was safer due to their efficacy and broad spectrum [12], led to antibiotics and copper compounds being set as the standard treatment for phytopathogenic bacteria [25,26]. The discovery of penicillin in 1928 started the antibiotic era, and these compounds enjoyed great success in the treatment of bacterial infections. However, due to their extensive application and mainly their abuse and inappropriate uses, the effectiveness of these compounds was drastically reduced due to the increase in antibiotic resistance in bacteria with the emergence of strains that are resistant to all known antibiotics [27,28]. This problem was further compounded with the appearance of phytopathogenic bacteria that are resistant to copper, as is the case for some *Pseudomonas* and *Xanthomonas* species [29,30].

The bacterial treatment predicament experienced all over the world since the 1980s, in particular the difficulty in obtaining novel antibiotics with the capacity to resolve current resistance problems [31], has resulted in a renewed interest in phage therapy [14]. In fact, phage therapy is one of the seven strategies highlighted by the US National Institute of Allergy and Infectious Diseases to address the problem of antibiotic resistance [16]. As indicated above, since 1924, phages have been applied as agents to eliminate phytopathogenic bacteria and are considered safe for use in agriculture for the control of plant diseases since the 1970s [32,33]. The evolution and the development of phages as biocontrol agents against plant pests have been the subject of several reviews since its inception in 1963 [34,35,36].

On the other hand, the quantity and the quality of agricultural crops can be also affected by the availability of pollinating insects. Insect pollination benefits up to 75% of plant species with importance in agriculture, and it is responsible for 35% of the world’s agricultural production. In this sense, and in addition to producing honey, bees are considered the most important pollinators. In particular, in the case of agricultural crops, 80% of pollination services are attributed to honeybees (*Apis mellifera*) [37]. This is why the decline of bee populations may be considered as a serious problem for the world’s agricultural production. This decline may be due to several reasons, including intensive farming practices, the use of chemical pesticides, and also because bees might be affected by different parasites and microorganisms. One of the most devastating bacterial diseases affecting bee larvae worldwide is American Foulbrood (AFB) caused by *Paenibacillus larvae*. Discarding the use of antibiotics for the reasons already mentioned, and because they could remain in the honey for a long time, thus compromising its quality and safety, incineration of the infected hives remains as the only viable treatment [38]. In this context, phage therapy also emerges as a promising alternative. For this purpose, different phages that have proven to be effective against numerous *P. larvae* strains [39,40,41] as well as promising endolysins [38] have been isolated and characterized.

In addition to agriculture, phages have also been considered for the control of infectious diseases in aquaculture [42,43]. As with other living organisms, fish in crowded conditions, such as those found in aquaculture, are susceptible to infection by microorganisms. Poor hygienic conditions in the pools used for farming often result in increased fish susceptibility to infection [44,45]. Diseases caused by bacteria constitute one of the most important problems in aquaculture, causing major economic losses [46]. Traditional treatment involves the use of antibiotics; however, this approach currently has limited success, partly due to the appearance of multi-drug resistant bacterial strains [47], and entails the added collateral risks [48]. All these factors make it imperative to find alternative methods of controlling bacterial diseases in aquaculture, with phage therapy in particular, as it has already demonstrated that it can be an effective therapy. The first successful report on the use of bacteriophages to combat pathogenic bacteria in aquaculture came from Japan and involved the bacterium *Lactococcus garviae* [49]. This success aroused great interest among researchers, who demonstrated that this approach could also be used against additional bacterial species, in particular those belonging to the genus *Vibrio* [42,45,50,51,52].

## 3. The Path to the Future

### 3.1. Agriculture

Plant pathogens are responsible for reducing the yield and the quality of agricultural products, causing large economic losses globally [53]. A variety of disease-causing pathogenic microorganisms, such as viruses, bacteria, and fungi, can infect plants, including economically-important agricultural crops [54]. According to Mansfield et al. 2012 [55], *Pseudomonas* spp., *Xanthomonas* spp., *Erwinia* spp., *Ralstonia* spp., *Agrobacterium* spp., *Xylella* spp., *Pectobacterium* spp., and *Dickeya* spp. constitute the most common genera of pathogenic bacteria.

Copper-based bactericides and antibiotics have traditionally been the main compounds used to treat plant diseases [56]. However, these treatments are not free of adverse side effects while currently displaying limited efficacy. From an environmental point of view, copper has the disadvantage of accumulating in the soil, which becomes toxic for both plants and animals, including humans [57]. On the other hand, antibiotics are not specific and harm both phytopathogenic and beneficial bacteria present in the environment [58], including beneficial microorganisms associated with plants. In addition, copper has been extensively used since 1880, and its effectiveness is declining; copper resistance, mediated by either a plasmid or chromosomal genes, has been reported in a variety of phytopathogenic bacteria [59,60,61]. Similarly, resistance to the antibiotic streptomycin (used in agriculture since 1950) was reported in a variety of bacterial species, including pathogenic strains of *X. versicatoria*, that infects tomatoes and peppers [62], and *E. amylovora,* an apple pathogen [63]. The extensive use of antibiotics not only in agriculture but also in the treatment of humans and animals has considerably contributed to the increased emergence of antimicrobial resistant (AMR) bacteria [64,65] as well as to the dissemination of the genes responsible for this antibiotic resistance (resistome). The spread of this resistome was facilitated by the rhizospheric microbiome [66]. The ever-growing number of AMR bacteria has resulted in a major reduction in the effectiveness of antibiotics, not only in agricultural settings but also in human health, with increasing numbers of human pathogens becoming resistant to current treatments, resulting in an escalation in mortality and morbidity from infectious diseases [64,65].

Due to the problems described above, there is currently a progressive trend to reduce the use of toxic compounds and antibiotics as pesticides, with the expectation that the use of some of these compounds may be banned in the future [67]. Furthermore, there is a widespread growing concern among consumers concerning the use of chemicals and antibiotics in the food growing industry, with some people already rejecting food produced using these products [68]. In summary, there is growing need and demand from both industry groups and consumers for new non-toxic, environmentally friendly pesticides with biological control, as part of an integrated pest management (IPM), as the most accepted alternative [54,69]. This highlights the role of phages as biological agents in the fight against phytopathogenic bacteria.

As a consequence, the last 20 years, and the last decade in particular, have seen a resurgence in phage research, with numerous studies focusing on the potential for phages to control bacterial plant diseases. A selection of some of the most relevant publications on the characterization and/or the efficacy (in vitro and/or in the field) of different phages against pathogens affecting diverse agricultural crops is summarized in Table 1.

#### 3.1.1. Potato Diseases

The most significant bacterial diseases affecting agricultural potato cultivation are soft rot and blackleg (caused by *Pectobacterium* and *Dickeya* species), bacterial wilt (resulting from *R. solanacearum* infection), and common scab (with *Streptomyces scabies* as the pathogen). Different phages and phage cocktails that are efficient against these diseases are shown in Table 1. These include the phage cocktail, used by Carstens et al. in 2018 [76] against soft rot caused by *D. solani*, which partially reduced the incidence (from 93% to 49%) and the severity (by 75%) of the disease. The bioassay carried out with the phages ΦPD10.3 and ΦPD23.1 to combat soft rot caused by *D. solani* and *Pectobacterium* species [73] resulted in an 80–95% reduction in the severity of the disease. It is worth mentioning that treatment with phage Wc5r proved effective against phage-resistant strains of *P. carotovorum* [77]; while phage ΦAS1 is an efficient therapy for potato common scab, produced by *S. scabies*, when used to treat seed tubers [70]. The study by Wei and colleagues, using a phage cocktail to combat the potato bacterial wilt caused by *R. solanacearum*, resulted in 80% of the plants protected against the bacterial pathogen [74].

#### 3.1.2. Tomato Diseases

Bacterial spot, produced by *X. campestris* pv. *Vesicatoria,* and bacterial wilt, caused by *R. solanacearum*, are the most important bacterial diseases affecting cultivated tomatoes. Different phages and phage cocktails have been successfully used to treat these plant diseases, as summarized in Table 1. These include a phage cocktail that reduced the severity of bacterial spot produced by *X. campestris* pv *vesicatoroa* by 17% in field experiments, which was also an effective (reduction of 40.5% to 0.9%) treatment for the disease in greenhouse conditions [78]. In other field experiments, the combination of phages with the acibenzolar-S-methyl (ASM) plant activator resulted in a more efficient control of bacterial spot as compared to the standard treatment combining copper and Mancozeb [80]. Also noteworthy is the research by Bae et al., demonstrating that phage PE204 (propagated using the host strain SL341) completely inhibited the tomato bacterial wilt disease [82].

#### 3.1.3. Additional Agricultural Crops

A variety of phages and/or combination of phages have also been effectively used against plant diseases, such as the radish common scab caused by *S. scabies* [83], the onion leaf blight resulting from *X. axonopodis* pv. *allii* infection [84], the lettuce soft rot caused by *P. carotovorum* ssp. [85], the bacterial blight in leeks infected with *P. syringae* pv. *porri* [86], and the leaf blight disease (BLB) produced in rice by *X. oryzae* [87], as summarized in Table 1. In addition, treatment of the cultivated mushroom *Pleurotus ostreatus* with phage ΦPto-bp6g resulted in effective protection of the fungi against *P. tolaasii* [94].

#### 3.1.4. Fruit Trees

A variety of commercially-important fruit tree diseases have been successfully treated with a variety of either phages or phage cocktails, as summarized in Table 1. These include grapefruit Asiatic citrus canker caused by *X. axonopodis* pv. *citri*, the orange bacterial spot produced by *X. axonopodis* pv. *citrumelo*, the fire blight resulting from the infection of pear and apple trees with *E. amylovora*, Pierce’s disease in vines infected with *Xylella fastidiosa* subsp. *fastidiosa*, and kiwi canker disease caused by *P. syringae* pv. *actinidiae*. These include the phage cocktail used in greenhouse experiments, which reduced the severity of grapefruit Asian citrus canker by 59% [88] and the phage cocktail used against the bacterial spot produced in oranges by *X. axonopodis* pv. *citrumelo*, shown in two trials to reduce the disease by 35–48% [88]. In addition, phages ΦEa1337-26 and ΦEa2345-6 reduced *E. amylovora* infection, which causes fire blight in pear trees, by 84% and 96%, respectively [89]. Additionally, the levels of *X. fastidiosa* (which causes the Pierce’s disease in grapes) were significantly reduced by inoculation with a cocktail consisting of four phages [90]. Furthermore, treatment with phages KHUΦ34, KHUΦ38, and KHUΦ44 produced a very effective lytic activity against different *P. syringae* pv. *actinidiae* (Psa) biovars responsible for kiwi canker disease; the effectiveness of the treatment changed according to the bacteriophages used [91]. In addition, the commercially available phage Φ6 was also effective, both in vitro and ex vivo, particularly against two highly aggressive Psa strains [92]. Similarly interesting, in a recent study, it has been shown that the combination of phages PN05 and PN09 and the natural antimicrobial carvacrol (2.0 mg/mL) controlled Psa regrowth for more than 40 h, preventing the emergence of phage-resistant mutants and controlling biofilm development [93].

### 3.2. Aquaculture

Commercial aquaculture has progressively become one of the main sectors involved in animal production, with a major role in human diets. Approximately 50% of fish and shellfish consumed by humans comes from aquaculture (FAO, Rome, Italy, 2016) [95]. However, the development and the growth of this industry has always been limited by infectious diseases affecting animals due to the high density and the homogeneity of the fish farmed. Despite the preventive measures progressively adopted, the stress produced on the animal populations by the high density in conjunction with potentially deficient hygiene measures and environmental deterioration provide conditions that favor emergence, propagation, and prevalence of infections, causing major economic losses [45]. The situation is further complicated by the fact that some of the fish pathogens can also cause important disease in humans [96]. As is the case for other the animal production industries, antibiotics constitute an integral part of fish management and are used in aquaculture both as prophylactics and for the treatment of bacterial infections [48]. Antibiotic therapy is currently experiencing conflicting effectiveness in aquaculture and, as discussed above, the selective pressure created by overuse and abuse of these compounds has also contributed to the selection and the spread of antibiotic-resistant bacteria in aquaculture conditions [97,98]. These difficulties, together with the warning by the World Health Organization (WHO) regarding antibiotic resistance [99], have galvanized the industry into the exploration of novel pathogen control alternatives. Although vaccination is an option for the control of infectious diseases, there are not many current vaccines authorized to use in aquaculture; furthermore, this approach is not feasible for crustaceans and mollusks, and its efficacy is either low or null for juvenile fish [100]. This makes it imperative to evaluate new options or complimentary alternatives for prevention and biocontrol of infectious diseases in aquaculture; these include the use of probiotics, phytobiotics, quorum sensing interference mechanisms, and particularly phage therapy [101,102].

Some bacterial species belonging to the genera *Edwardsiella*, *Lactococcus*, *Pseudomonas*, *Aeromonas*, and *Flavobacterium*, but mainly to the genus *Vibrio*, constitute the main bacterial pathogens of cultured fish and shellfish. Numerous in vitro assays testing the effect of phage therapy on fish pathogenic bacteria have been carried out over the last two decades; in addition, a number of in vivo studies have also evaluated the potential of bacteriophages for controlling bacterial infections in aquaculture. These studies include the use of phages to effectively combat multidrug resistant bacteria [103,104]. A selection of some of the most relevant studies on the characterization and/or the efficacy (in vitro and/or in the field) of phages from different families against a wide range of pathogens that cause a variety of disease in many fish and shellfish species are shown in Table 2. These include the first studies describing the protective effect of phage PLgY-16 (administered either orally or intraperitoneally) against lactococcosis, a disease of yellowtail (*Seriola quinqueradiata*) infected with *L. garvieae* [49]. It is also worth highlighting the use of phage PPpW-4, administered in the feed, to combat the bacterial hemorrhagic ascites disease in ayu fish (*Plecoglossus altivelis*) [105] caused by *P. plecoglossicida*. Many of the diseases caused by different species belonging to the genus *Vibrio* have also been efficiently controlled by phages. In particular, treatment of *Penaeus monodon* larvae suffering from luminescent vibriosis produced by *V. harveyi* with phages from the *Siphoviridae* family (the bacteriophages were added in the tank water) resulted in an 85% survival of the larvae, as compared to 65–68% of animals surviving after antibiotic treatment [106].

Table 2 also includes an example of a lytic phage, also applied in the tank water, that was successfully used to treat Atlantic salmon (*Salmo salar*) infected with *V. anguillarum*; the treatment resulted in a survival rate of up to 100%, while lessthan 10% of the untreated fish survived the disease [113]. It is also noteworthy to mention that a cocktail of phages belonging to the family *Siphoviridae* and applied in the water effectively controlled the vibriosis produced by *Vibrio* sp. Va-F3 in the shrimp *Litopenaeus vannamei*, increasing its survival from 20% (untreated group) to 91.4% (phage treated group); the success rate of the treatment was equivalent to that obtained with antibiotic therapy [116]. Phage treatment also proved effective against a variety of additional fish pathogens, with the administration by intramuscular injection of phages Φ2, and Φ5 (*Myoviridae*) achieving up to 100% survival in catfish (*Pangasianodon hypophthalmus*) infected with Motile Aeromonas Septicemia (MAS), caused by *A. hydrophila*; the survival rate was 18.3% in untreated fish [118]. A similar remarkable protective effect was achieved with the PAS-1 phage when used to combat the furunculosis caused by *A. salmonicida* in Rainbow trout (*Oncorhynchus mykiss*) [119]. In in vitro studies, the use of effective phage cocktails (AS-A, AS-D, AS-E) against this pathogen showed a faster control of bacterial concentration as well as a decrease in the frequency of occurrence of phage-resistant mutants. [122]. Worthy of note is also the success obtained when the phage FCP1 was administered intramuscularly to treat the disease caused by *F. columnaris* in the catfish *Clarias batrachus* [126].

## 4. Challenges to Be Address in Phage Therapy

As pointed out above, phages constitute a very promising alternative for the treatment of bacterial diseases in both agriculture and aquaculture. However, the use of phages still presents a number of challenges that need to be thoroughly investigated in order to make their use a reality (summarized in Box 2).

Box 2 **Phage therapy challenges to be addressed.**

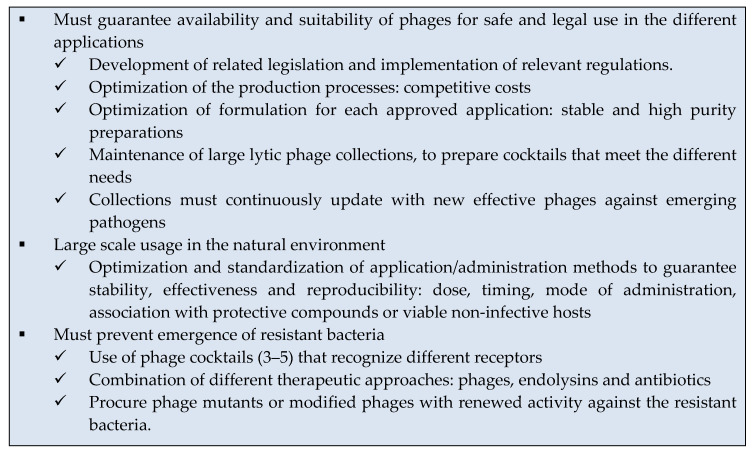

One important hurdle deals with the use of bacteriophages on a large scale in the natural environment. One of the major problems in agriculture is the potential instability of phages in the environment in both plant microbial habits, phyllosophere and rhizosphere, due to the effect of external factors such as temperature, desiccation and, in particular, UV light. This highlights the importance of optimizing the phage application time or season and the question of how to best administer the bacteriophage accompanied by a viable host that is harmless to the plant or protected by non-infectivity affecting compounds [79,127,128]. The challenges in aquaculture include the inability of some phages to reproduce in the environment, even when their lytic cycle is well defined and studied under in vitro conditions [129]. In addition, the advantages and the disadvantages of different methods of large scale administration, whether by injection, as a food additive, or externally by immersion, must be evaluated, not only for each pathogen and disease but also according to the development stage of the animal, in order to determine the best standards for their use and draw contingency plans for future treatment [50,130,131].

Another important point refers to the availability and the suitability of phages as safe treatments in different applications. Given the specificity of phages that often only infect one or a few bacterial strains, it is essential to develop large collections of lytic bacteriophages in order to prepare customized cocktails that can infect the pathogenic bacteria [132]. In addition, these collections must be continuously updated to include novel phages that are effective against emerging bacterial strains. These requirements can only be met by increasing the bacteriophage research, not only to obtain novel phages but also to fully understand their molecular biology and mechanisms of action. The accumulated knowledge will also help eliminate potential risks related to the use of phages; these include the possibility of transferring virulence or other harmful genes as well as any putative unwanted effects they could exert on other microorganisms that are either part of the microbiota associated with treated plants and animals or present in the environment [133].

Another aspect that needs extensive research deals with the optimization of the production processes; these procedures must not only guarantee the production of a great variety of phages, but it is also imperative that they do so at competitive pricing [134]. Another area that cannot be forgotten is the optimization of the formulation of the phage cocktails that must meet the needs of each application while guaranteeing stability and high purity of the preparations, which must be free from contaminants such as endotoxins or lipopolysaccharides (LPS) [135].

In addition, legislation on bacteriophage use must be developed in parallel to the above mentioned optimization in application and large scale production of phages; this legislation is the responsibility of governments that need to implement the relevant regulations for phage therapy under legal conditions and sanitary environment security [136].

The last and most worrisome aspect relates to the already described emergence of phage-resistant bacteria [137], and this matter could become one of the major limiting factors in the use of phages for the control of bacterial infections. Resistance can be acquired by either mutation and selection and/or by horizontal gene transfer, and this characteristic can be transferred to the descendants [138,139]. All six stages of phage infection (attachment, penetration, transcription, biosynthesis, maturation, and lysis) are susceptible to the development of resistant bacteria. The mechanisms involved in this resistance include prevention of phage adsorption, blocking DNA entry, abortive infection, and the role played by CRISPR/Cas and modification-restriction systems [140]. However, studies conducted have thus far indicate that bacterial resistance to phages is around 10 times lower than antibiotic resistance [141]. An additional advantage is that, unlike antibiotic resistance, phage resistance produces a less virulent microorganism, as phage receptors on the surface of the bacteria also act as virulence factors [142,143]. This attenuation of virulence was observed in bacterial strains from species such as *F. columnare* [144], *F. psychrophilum* [145], and *V. anguillarum* [146].

The emergence of phage resistant bacteria has been reported not only in plant pathogens such as *E. carotovora* [147] and *P. atrosepticum* [148] but also in fish pathogens such as *P. plecoglossicidae* [149], *A. salmonicida* [120], and *Streptococcus iniae* [150]. As is the case for the resistance to antimicrobial agents, this problem can be prevented or resolved by the use of different strategies. These include applying cocktails containing not just a single phage but a mixture of three to five different phages [36,151] and the use of phage endolysins [8,104] in order to prevent the appearance of resistance during phage treatments. Bacterial resistance to phages usually appears at a rate between 10^−6^ and 10^−8^. Infection with a mixture of bacteriophages reduces the rate of occurrence of resistances in a variable range (10^−4^–10^−8^ for most of the studies). The highest reductions are indeed achieved with cocktails that include several phages exhibiting different routes of infection. [96,122,152]. Additional strategies to prevent and combat the emergence of microbial resistance include the combination of different therapeutic approaches, the use of mutant phages obtained from the wild type bacteriophage that regain their activity against the bacteria [140], and the isolation of novel or modified phages [153] that are effective against the resistant microorganisms.

## 5. Conclusions

In conclusion, phage therapy was rediscovered two decades ago to counteract the current difficulties posed by bacterial resistance to antibiotics and, in particular, the recent appearance of bacterial strains that are not only multi-resistant but can overcome treatment with all known antibiotics. Phage therapy has demonstrated its potential effectiveness in the prevention and the control of important bacterial infections, both in agriculture and aquaculture, and it has proved to represent an excellent and viable alternative to antibiotic treatment. Unfortunately, although phages were discovered more than one hundred years ago, phage research was eclipsed by the dominant role given to antibiotics in human and animal health; these compounds were hailed as “silver bullets” that could combat any human or animal diseases. This short-sighted approach truncated the development of a solid phage research in addition to considerably limiting the effort to investigation alternative approaches to antibiotic treatment. This lack of research diversity has resulted in the conundrum we are facing now, with antibiotic resistant rampant in bacteria and no viable short-term alternative to combat pathogenic organisms. It is essential to immediately rectify and direct research efforts and money into areas such as phage therapy, which have already demonstrated a great potential in the control and the elimination of bacterial pathogens. This promising technique needs increased research efforts in order to design effective and reproducible treatments that need to be customized for the different applications. In addition, it is also required that governments and organizations develop the relevant legislation to guarantee appropriate and safe use of these technologies. Finally, we must learn from the mistakes of the past in order to prevent drawbacks and problems in the treatment of pathogenic bacteria. With this in mind, we propose the use of phage cocktails as biocontrol agents in agriculture and aquaculture, combined with the use of endolysins and antibiotics within the framework of an integrated microbial infections management, to prevent the appearance of resistant bacterial strains.

## Figures and Tables

**Table 1:** Relevant examples of phages used in the biocontrol of plant pathogens.

Phage/Phages Cocktails (Family)	Target Microorganism	Plant	Disease	Relevant Achievements	Reference
ΦAS1 (*Siphoviridae*)	*Streptomyces scabies*	Potato	Common scab	Isolation of a new phage from a potato field near Albany, Western Australia Phage propagation by an effective mini-biorreactor Efficient disinfection of seed potato tubers: phage-treated seeds significantly reduced the levels of surface lesions of scab compared with untreated tubers	[70]
vB_DsoM_LIMEstone1, vB_DsoM_LIMEstone2 (*Myoviridae*)	*Dickeya solani*	Potato	Soft rot/Blackleg	Isolation of new phages from soil samples from a potato field trial (Merelbeke, Belgium) In laboratory assays, the phages reduced the disease incidence and the severity on potato tubers. In a field trial (using potato tubers infected with *D. solani*), the phage treatment resulted in higher crop yields	[71]
ΦD1, ΦD2, ΦD3, ΦD4, ΦD5, ΦD7, ΦD9, ΦD10, ΦD11 (*Myoviridae*)	*Dickeya solani*	Potato	Soft rot/Blackleg	Isolation of new phages from soil samples collected in different regions in Poland In the in vitro and potato slice assay experiments, phages were able to stop the growth and efficiently lyse *D. solani* cells, protecting the tuber tissue from maceration caused by the bacteria	[72]
ΦPD10.3, ΦPD23.1 (*Myoviridae*)	*Pectobacterium carotovorum ssp*. *carotovorum* *P. wasabiae* *Dickeya solani*	Potato	Soft rot/Blackleg	Isolation of new phages from soil, rhizosphere soil, and potato plant and tuber samples obtained from different regions in Poland Phage genomes were characterized and proteomes obtained In potato slice and whole tuber assays, the phages (applied individually or together) were able to reproducibly and significantly reduce soft rot infections when compared to controls (inoculated with a mixture of bacteria)	[73]
P-PSG-1 (*Siphoviridae*), P-PSG-2, P-PSG-3, P-PSG-7 (*Siphoviridae*), P-PSG-8, and P-PSG-9	*Ralstonia solanacearum*	Potato	Bacterial wilt	Isolation of new phages from different water sources in Kenya and China A phage cocktail with speed and efficacy in the lysis of *R. solanacearum* enhanced was formulated The phage cocktail was very effective protecting the potato plants from bacterial wilt by injection of the phages into the plants. The cocktail was also able to reduce the density of live bacteria in pathogen-contaminated sterilized soils	[74]
vB_PatP_CB1, vB_PatP_CB3, vB_PatP_CB4 (*Podoviridae*)	*Pectobacterium atrosepticum*	Potato	Soft rot/Blackleg	Isolation of new phages from soil samples collected from potato grading machinery and potato fields from two farms in Co. Cork, Ireland Phage genomes were characterized and the proteome for CB1 obtained The cocktail containing phages CB1, CB3, and CB4 showed effective protective effect, evaluated on whole tubers, against the infection caused by a mix of two strains of *P. atrosepticum* (DSM 18077 and DSM 30186)	[75]
Dagda, Dagda_B1, Katbat, Luksen, Mysterion, P694 (*Podoviridae*)	*Dickeya solani*	Potato	Soft rot/Blackleg	Isolation of new phages from different environments at different time points and at different locations in Denmark A phage cocktail formulated with ability to significantly decrease the incidence of soft rot and the disease severity after 5 days of storage post-infection with *D. solani*	[76]
Wc5r, Phage cocktail	*Pectobacterim atrosepticum* *P. carotovorum*	Potato	Soft rot/Blackleg	Isolation of new phages from soil and water samples collected in Wuhan, China Phage Wc5r showed cross-activity against *P. atrosepticum* and two phage-resistant *P. carotovorum* strains A formulated phage cocktail applied on potato slices (before or within an hour after bacterial inoculation) was able to reduce 90% soft rot symptoms	[77]
Mixture of four h-mutant (Agriphage, Agriphi, Logan, Utah)	*Xanthomonas campestris pv. vesicatoria*	Tomato	Bacterial spot	Foliar applications of phages to field-grown tomatoes decreased disease severity compared with untreated control plants. The incidence of bacterial spot on greenhouse-grown seedlings was also reduced in bacteriophage-treated plants	[78]
Formulated phage cocktails	*Xanthomonas campestris pv. vesicatoria*	Tomato	Bacterial spot	The formulations analyzed significantly increased the longevity of the phage on the plant surface Protective formulations significantly improved the efficacy of phage treatments both in the greenhouse and in the field. Skim milk and Casecrete gave the best results in greenhouse and field assays, respectively	[79]
6 Phages (Agriphage, OmniLytics, Inc., Salt Lake Cith, UT) combined with plant activator (ASM)	*Xanthomonas campestris pv. vesicatori*a	Tomato	Bacterial spot	Formulation of a phage mixture containing six different phages specific to *X. campestris pv. vesicatoria* race T3 strain 91–118 In field experiments, application of host-specific phages was effective against the bacterial spot pathogen, showing better disease containment than with copper-Mancozeb or the untreated controls	[80]
ΦRSL1 (*Myoviridae*)	*Ralstonia solanacearum*	Tomato	Bacterial wilt	Alternative phage biocontrol method using a unique phage instead of a phage cocktail During the experimental period, tomato plants treated with ΦRSL1 phage showed no symptoms of wilting, whereas all untreated plants had wilted by 18 days post-infection. Active ΦRSL1 particles can be recovered from the roots of treated plants and from soils 4 months post-infection	[81]
PE204 (*Podoviridae*)	*Ralstonia solanacearum*	Tomato	Bacterial wilt	Simultaneous application of phage PE204 and *R. solanacearum* on tomato rhizosphere completely inhibited the occurrence of bacterial wilt. Addition of Silwet L-77 to the phage suspension did not impair the disease control activity of the phage, allowing the control of the bacterial wilt	[82]
Stsc1, Stsc3 (*Siphoviridae*)	*Streptomyces scabies*	Radish	Common scab	Isolation of new phages from soil samples Phages Stsc1 and Stsc3 infected 88% and 75% of the pathogenic *S. scabiei* strains tested, respectively Both phages prevented symptoms of *S. scabiei* on radish seedlings	[83]
Bacteriophage mixture (AgriPhage, OmniLytics, Salt Lake City, UT)	*Xanthomonas axonopodis pv. allii*	Onion	*Xanthomonas* leaf blight	Under field conditions, applications of the mix of phages reduced disease severity in an equivalent or better manner than weekly applications of copper hydroxide plus Mancozeb. Phage populations remained on onion leaves for at least 72 to 96 h under field and greenhouse conditions, respectively	[84]
PP1 (*Podoviridae*)	*Pectobacterium carotovorum ssp. carotovorum*	Lettuce	Soft rot/Blackleg	Isolation of a new phage from soil samples (Chinese cabbage fields) A rapid and strong lytic activity against its host bacteria was shown by the new phage Treatment with phage PP1 significantly reduced the disease caused by *P. carotovorum subsp. carotovorum*	[85]
vB_PsyM_KIL1, vB_PsyM_KIL2, vB_PsyM_KIL3, vB_PsyM_KIL4, and vB_PsyM_KIL5, vB_PsyM_KIL3b (*Myoviridae*)	*Pseudomonas syringae pv. porri*	Leek	Bacterial blight	Isolation of new phages from infected fields in Flanders Phage genomes were characterized and proteomes obtained Classified into a novel clade Development of a phage cocktail effective against 41 tested strains Specific bio-assays showed the in planta effectiveness of phages and attenuation of symptoms development in a field experiment	[86]
ΦXOT1, ΦXOT2, ΦXOM1, ΦXOM2, ΦXOF1, ΦXOF2, ΦXOF3, ΦXOF4 (*Siphoviridae*)	*Xanthomonas oryzae*	Rice	Leaf blight disease (BLB)	Isolation of new phages from diseased plant leaves and soil samples The phage ΦXOF4 showed the broadest host range, killing all the pathogenic *X. oryzae* strains tested Seedlings raised from phage-treated seeds displayed complete bacterial growth inhibition and reduced incidence of BLB disease	[87]
CP2, ΦXac2005-1, ccΦ7, ccΦ13, ΦXacm2004-4, ΦXacm2004-16, ΦX44, ΦXaacA1	*Xanthomonas axonopodis pv. citri*	Grapefruit	Asiatic citrus canker	In greenhouse assays, phage treatment significantly reduced the disease severity when applied without skim milk. If skim milk was added, no disease reduction was observed In a citrus nursery no synergistic effect was observed by combining phages and copper-Mancozeb	[88]
ΦEa1337-26 (*Podoviridae*), ΦEa2345-6 (*Myoviridae*)	*Erwinia amylovora*	Pear and apple trees	Fire blight	Isolation of new phages from apple and pear orchards in the Okanagan and Fraser Valleys of British Columbia Phages ΦEa1337-26 and ΦEa2345-6 reduced the bacterial infection by 84% and 96%, respectively, when applied on detached pear blossoms using as a carrier the epiphyte bacterium *Pantoea agglomerans* Eh21-5 Phage ΦEa2345-6, combined with Eh21-5, reduced infection of fire blight on apple flowers of potted apple trees with an efficacy comparable to the antibiotic streptomycin	[89]
CP2, ΦXac2005-1, ccΦ7, ccΦ13, ΦXacm2004-4, ΦXacm2004-16, ΦX44, ΦXaacA1	*Xanthomonas axonopodis pv. citrumelo*	Orange	Citrus bacterial spot	In a commercial citrus nursery, phages application significantly reduced the progress of the disease on Valencia oranges (a moderately susceptible culture) In an experimental nursery, under low disease pressure, phage treatment significantly reduced the disease incidence providing similar levels of control than copper-Mancozeb treatment	[88]
Sano, Salvo, Prado, Paz	*Xylella fastidiosa subsp. fastidiosa*	Grapevines	Pierce’s disease (PD)	Development of a cocktail with four lytic phages The phage cocktail significantly reduced *X. fastidiosa* levels in grapevines and prevented the development of PD symptoms No in planta phage-resistant *X. fastidiosa* isolates were recovered, and in vitro selected *X. fastidiosa* mutants did not cause PD symptoms	[90]
KHUΦ34 (*Myoviridae*), KHUΦ38 (*Podoviridae*), KHUΦ44 (*Myoviridae*)	*Pseudomonas syringae* pv*. actinidiae*	Kiwifruit	Canker disease	Isolation of new phages from soils collected from kiwifruit orchards in South Korea Phages showed activity against strains of *P. syringae* pv. *actinidiae*, including Psa2 and Psa3 groups. Some of them were also effective against other *P. syringae* pathovars The effective lytic activity of phages KHUΦ34, KHUΦ38, and KHUΦ44 allows one to propose their potential use in the control of bacterial canker disease in kiwifruits	[91]
Φ6 (*Cystoviridae*) Leibniz- Institute DSMZ collection	*Pseudomonas syringae* pv*. actinidiae*	Kiwifruit	Canker disease	Phage Φ6 was effective against two biovar 3 (a highly aggressive pathogen) Psa strains using both in vitro and ex vivo test The inactivated CRA-FRU 14.10 Psa strain did not re-grow after treatment was concluded	[92]
PN05 PN09 Cocktail of both phages (*Myoviridae*)	*Pseudomonas syringae* pv*. actinidiae*	Kiwifruit	Canker disease	Isolation of new phages from water samples collected in Hangzhou, China The combined treatment with phages (PN05 and PN09) and carvacrol effectively reduced the Psa concentration, preventing the emergence of phage-resistant mutants and controlling biofilm development	[93]
ΦPto-bp6g	*Pseudomonas tolaasii*	*Pleurotus ostreatus*	Brown blotch disease	The phage ΦPto-bp6g was characterized at the genomic level Phage ΦPto-bp6g showed strong bactericidal activity against *P. tolaasii* The mushroom (*P. ostreatus*) buds treated with the mixture of *P. tolaasii* and phage ΦPto-bp6g, exhibited over time the same growing pattern that the control, developing normal mushroom fruit bodies	[94]

Entries are grouped according to the agricultural crop, and the works related to the same crop were ordered chronologically.

**Table 2:** A selection of phages reported for the biocontrol of pathogens in aquaculture.

Phage/Phages Cocktails (Family)	Target Microorganism	Fish or Aquaculture Product	Disease	Relevant Achievements	Reference
PLgY-16, PLgY-30, PLgW-1 (*Siphoviridae*)	*Lactococcus garvieae*	Yellowtail (*Seriola quinqueradiata*)	Lactococcosis	Isolation of new phages: PLgY-16 and PLgY were obtained from cultures of *L. garviae* isolated from diseased yellowtail; PLgW-1 was isolated from natural seawater According to the results, anti-*L. garvieae* phage (administered orally or intraperitoneally) protected fish from experimental *L. garvieae* infection	[49]
PPpW-3 (*Podoviridae*) PPpW-4 (*Myoviridae*) and a mixture of PPpW-3/W-4	*Pseudomonas plecoglossicida* PTH-9802 strain	Ayu fish (*Plecoglossus altivelis*)	Bacterial hemorrhagic ascites disease	Phages inhibited in vitro the growth of *P. plecoglossicida*. The highest inhibitory activity was shown by the mixture In a field trial, when phage PPpW-3/W-4 was supplied with the food to ayu in a pond where the disease occurred naturally, fish mortality decreased to one-third after a 2 week period	[105]
Viha8, Viha10 (*Siphoviridae*) Viha9, Viha11	*Vibrio harveyi*	Shimp larvae (*Penaeus monodon*)	Luminescent vibriosis	Isolation of new phages: three from oyster tissue and one from shrimp hatchery water Phage Viha10 was effective in reducing the population of *V. harveyi* in the biofilm formed on a high density polyethylene surface In hatchery trials, the application of phage treatment (Viha8 and Viha10) resulted in 85% survival of *P. monodon* larvae	[106]
Viha 1, Viha 2, Viha 3,Viha 5, Viha 6, Viha 7 (*Siphoviridae*) Viha4 (*Myoviridae*)	*Vibrio harveyi*	*Penaeid shrimp*	Luminescent vibriosis	Isolation of new phages from shrimp farms, hatcheries, and tidal creeks along the east and the west coasts of India Phages were found to be highly lytic for *V. harveyi*. Six of them had a broad lytic spectrum, thus they could be potential candidates for biocontrol of this bacterium in aquaculture systems	[107]
VhCCS-01, VhCCS-02, VhCCS-04, VhCCS-06, VhCCS-17, VhCCS-20 (*Siphoviridae*) VhCCS-19, VhCCS-21 (*Myoviridae*)	*Vibrio harveyi*	Phyllosoma larvae of the tropical rock lobster (*Panulirus ornatus*)	Luminescent vibriosis	Isolation of new phages from water samples from discharge channels and grow-out ponds of a prawn farm in northeastern Australia The host range for purified phage included *V. harveyi*, *V. campbellii*, *V. rotiferianus* and *V. parahaemolyticus* The lytic ability of the isolated phages suggested that they are appropriate for phage therapy	[108]
vB_VhaS-a, vB_VhaS (*Siphoviridae*)	*Vibrio harveyi*	Abalone (*Haliotis laevigata*).	Vibriosis	Isolation of new phages from water or tissue sample liquid In in vitro assays, the phages showed different antimicrobial abilities towards different *V. harveyi* isolates In the bioassay, the treatment with phage resulted in 70% of abalone survival, as compared to the 0% exhibited by the positive bacterial control	[109]
pVp-1 (*Siphoviridae*)	*Vibrio parahaemolyticus*	Oysters	Luminescent vibriosis	Both by bath immersion and oyster surface-application, the lytic phage effectively reduced the bacterial growth of a multiple-antibiotic-resistant *V. parahaemolyticus* pandemic strain	[110]
vB_VpS_BA3, vB-VpS_ CA8 (*Siphoviridae*)	*Vibrio parahaemolyticus*	-	-	Isolation of new phages from sewage collected in Guangzhou, China Taking into account the determined host range and the rate of inactivation in the in vitro phage-killing assay, phages, particularly CA8, had the potential to be used in phage therapy	[111]
ΦVP-1 (*Myoviridae*)	Multiple-drug-resistat *Vibrio parahaemolyticus* and *Vibrio alginolyticus*	Penaeid shrimp	Antibiofilm activity	Isolation of a new phage from shrimp pond water samples collected from aquafarms in Cochin, India Ability to infect multiple-drug-resistant strains of mangrove and seafood origin belonging to the species *V. parahaemolyticus* and *V. alginolyticus*, and showing also biofilm reducing capacity	[112]
309, ALMED, CHOED, ALME, CHOD, CHOB	*Vibrio anguillarum*	Fish Atlantic salmon (*Salmo salar*)	Hemorrhagic septicemia	Isolation of new phages from bivalve samples purchased in the central market of Santiago, Chile The phages exhibited ability to infect both *V. anguillarum* and *V. ordalii* but not *V. parahaemolyticus* strains In both experimental tanks and fish farm assays, the CHOED phage was able to protect *Salmo salar* against experimentally induced vibriosis	[113]
vB_VspP_pVa5 (N4-like podovirus)	*Vibrio splendidus*	Fish and bivalves	Severe epizootics Skin Ulceration Syndrome (SUS)	Isolation of a new phage from a sea-cage aquaculture farm in Greece with a very specific host range infecting only the bacterial host The phage showed an intense bactericidal activity being proposed as a potential candidate for phage cocktails, suitable for the biological control of *V. splendidus*	[114]
pVco-14 (*Siphoviridae*)	*Vibrio coralliilyticus*	Pacific oyster larvae (*Crassostrea gigas*)	Massive mortality of Pacific oyster larvae	Isolation of a new phage from the sewage at the oyster hatchery located at Tongyeong, Korea that specifically infects *V. coralliilyticus* Oyster larvae pre-treated with phage pVco-14 before the bacterial challenge exhibited significantly higher survival rate when compared to the untreated groups	[115]
ValLY-3, VspDsh-1, VspSw-1, VpaJT-1, and ValSw4-1 (*Siphoviridae*)	*Vibrio sp.* Va-F3 strain	Shrimp (*Litopenaeus vannamei*)	Vibriosis	Isolation of new phages from wastewater samples collected from sewage draining exits in the cities of Shenzhen, Zhanjiang, and Shanwei, China A workflow of preparing a phage cocktail was described: the phage cocktail preparation showed in vitro higher efficiency in inhibiting the growth of *Vibrio* sp. Va-F3 than any single phage In in situ experiments, the survival rate of the group of shrimp treated with the cocktail was comparable to that of the group treated with antibiotics	[116]
Different bacteriophages	*Aeromonas hydrophila* and *Edwardsiella tarda*	Japanese eel (*Anguilla Japonica*)	Hemorrhagic septicaemia and edwardsiellosis	Isolation of new phages from water samples in southern Taiwan In pure culture, the phages decreased the bacterial host by three orders of magnitude after two hours In pond water, phage treatment reduced 250-fold the *A. hydrophila* population in 8 h, while phage population increased	[117]
Φ2, Φ5 (*Myoviridae*)	*Aeromonas hydrophila*	Catfish *(Pangasianodon hypophthalmus)*	Motile Aeromonas Septicemia (MAS)	Isolation of new phages from water samples from the Saigon River of Ho Chi Minh City, Vietnam Phages exhibited broad activity spectra, including multiple-antibiotic-resistant *Aeromonas* isolates Phage treatments applied to infected catfish resulted in a significant increase in the survival rates when compared to control experiments	[118]
Akh-2 (*Siphoviridae*)	*Aeromonas hydrophila*	Loach (*Misgurnus anguillicaudatu*)	Septicemia	Isolation of a new phage from water collected from Wahyeon Beach, Geoje Island, South Korea In an experiment where the disease was artificially induced, loach treated with phage Akh-2 exhibited an increased survival rate as compared with the untreated control	[119]
HER 110 (*Myoviridae*)	*Aeromonas salmonicida* HER 1107 strain	Brook trout *(Oncorhynchus* *fontinalis)* *formerly, (Salvelinus fontinalis)*	Furunculosis	In aquarium assays, treatment with phage HER 110 declined the population of *A. salmonicida* in 3 days and additionally, the onset of furunculosis in brook trout was delayed by 7 days. Different phages were active against *A. salmonicida* HER 1107. The mutants that developed resistance to phage HER 110 were sensitive to other phages	[120]
PAS-1	*Aeromonas salmonicida*	*Rainbow trout* (*Oncorhynchus mykiss*)	Furunculosis	The phage showed in vitro efficient bacteriolytic activity against *A. salmonicida subsp. salmonicida* strain AS05 In tank experiments, the administration of phage PAS-1 to *A. salmonicida subsp. salmonicida*-infected rainbow trout exhibited notable protective effects, increasing survival rates and mean times to death	[121]
AS-A AS-D AS-E Cocktails combining two or three phages	*Aeromonas salmonicida*	*-*	Furunculosis	Isolation of new phages from sewage network of Aveiro, Portugal Phage cocktails developed Phage cocktails reduced the population of *A. salmonicida* faster than single suspensions and decreased the development of phage-resistant mutants. Because of this, they were proposed to be used to control furunculosis in aquaculture	[122]
ETP-1 (*Podoviridae*)	Multidrug resistant *Edwardsiella tarda*	Zebrafish (*Danio rerio*)	Edwardsiellosis	Isolation of a new phage from marine fish farm water in Jeju Island, Korea Effective against multidrug-resistant *E. tarda* When zebrafish was bath exposed for 12 days to phage ETP-1, and simultaneously challenged with *E. tarda*, the survival rate in phage-exposed fish was higher than that found in the control until 4 days post challenge	[103]
ΦeiDWF, ΦeiAU, ΦeiMSLS (*Siphoviridae*)	*Edwardsiella ictaluri*	Catfish	Enteric septicemia	Isolation of new phages from geographically distant aquaculture ponds at different times According to the genomic analysis, the phages are members of a lineage highly stable over time and geographic regions. The genome analysis also revealed that the virus were virulent phages lacking lysogeny capacity, which will facilitate therapeutic applications	[123]
FpV-1 to FpV-22: FpV2, FpV4 (*Podoviridae*) FpV7, FpV9, FpV10 (*Siphoviridae*) FpV14, FpV19 (*Myoviridae*)	*Flavobacterium psychrophilum*	Rainbow trout (*Oncorhynchus mykiss*) and other species of trouts	Rainbow trout fry syndrome (RTFS) and bacterial coldwater disease (CWD)	Isolation of new phages from Danish rainbow trout farms, both with and without outbreaks of RTFS when the samples were taken The phages showed a broad-host-range with a strong lytic potential against a large number of pathogenic *F. psychrophilum* host strains, indicating that they could have potential in the treatment of RTFS and CWD	[124]
PFpW-3, PFpC-Y (*Myoviridae*) PFpW-6, PFpW-7 (*Podoviridae*) PFpW-8 (*Siphoviridae*)	*Flavobacterium psychrophilum*	Ayu fish (*Plecoglossus altivelis altivelis*)	Systemic bacterial coldwater disease (CWD)	Isolation of new phages from ayu kidneys and pond water collected from Japanese ayu farms Among the phages, in in vitro assays, PFpW-3 displayed high infectivity for *F. psychrophilum* isolated from ayu and from other fish, indicating that it could have interest for the treatment of CWD in Japanese ayu farms	[125]
FCP1–FCP9 FCP1 (*Podovariedae*)	*Flavobacterium columnare*	Catfish (*Clarias batrachus*)	Columnaris disease	Isolation of new phages from the water and the bottom sediments of various geo-climatic regions of North India When *C. batrachus* was treated with a virulent bacterial isolate and with phage FCP1 (applied via intramuscular, immersion, and oral), a significant decrease in host bacterium in sera, gill, liver, and kidney of challenged fishes was observed Phage treatment resulted in disappearance of gross symptoms and 100% survival in experimentally infected *C. batrachus*	[126]

Entries are grouped according to the pathogenic species, and the works related to the same species were ordered chronologically.
